# Effects of DPTQ, a novel positive allosteric modulator of the dopamine D1 receptor, on spontaneous eye blink rate and spatial working memory in the nonhuman primate

**DOI:** 10.1007/s00213-022-06282-7

**Published:** 2023-03-24

**Authors:** Stacy A. Castner, Linli Zhang, Charles R. Yang, Junliang Hao, Jeffrey W. Cramer, Xushan Wang, Robert F. Bruns, Hugh Marston, Kjell A. Svensson, Graham V. Williams

**Affiliations:** 1 Department of Comparative Medicine, Yale University, 310 Cedar St, New Haven, CT 06520 USA; 2 ChemPartner, 99 Lian He North Road, Zhe Lin Town, Fengxian Area, Shanghai, China; 3 Eli Lilly & Co, Lilly Corporate Center, Indianapolis, IN 46285 USA; 4 Eli Lilly & Co, Erlwood, Sussex UK

**Keywords:** Positive allosteric modulator, Dopamine, D1, Eye blink, Working memory, Spatial delayed response, Cyclic AMP, Plasticity

## Abstract

**Rationale:**

Dopamine (DA) signaling through the D1 receptor has been shown to be integral to multiple aspects of cognition, including the core process of working memory. The discovery of positive allosteric modulators (PAMs) of the D1 receptor has enabled treatment modalities that may have alternative benefits to orthosteric D1 agonists arising from a synergism of action with functional D1 receptor signaling.

**Objectives:**

To investigate this potential, we have studied the effects of the novel D1 PAM DPTQ on a spatial delayed response working memory task in the rhesus monkey. Initial studies indicated that DPTQ binds to primate D1R with high affinity and selectivity and elevates spontaneous eye blink rate in rhesus monkeys in a dose-dependent manner consistent with plasma ligand exposures and central D1activation.

**Results:**

Based on those results, DPTQ was tested at 2.5 mg/kg IM in the working memory task. No acute effect was observed 1 h after dosing, but performance was impaired 48 h later. Remarkably, this deficit was immediately followed by a significant enhancement in cognition over the next 3 days. In a second experiment in which DPTQ was administered on days 1 and 5, the early impairment was smaller and did not reach statistical significance, but statistically significant enhancement of performance was observed over the following week. Lower doses of 0.1 and 1.0 mg/kg were also capable of producing this protracted enhancement without inducing any transient impairment.

**Conclusions:**

DPTQ exemplifies a class of D1PAMs that may be capable of providing long-term improvements in working memory.

## Introduction

Dopamine (DA) is critically involved in many aspects of the brain function including motor activity, wakefulness, and cognition. The D1 receptor in particular is crucial to maintaining high-order cognitive functions in the primate, including attention, executive function, and working memory, and this receptor is abundant in primate prefrontal cortex where its signaling is integral to the core cognitive process of working memory (Sawaguchi and Goldman-Rakic 1991; Goldman-Rakic et al. 2004). Working memory is a key indicator of outcome in patients with schizophrenia and its function becomes diminished in aging and neurodegenerative disorders (Bodnar et al. 2008; Rhode and Katz 2017; Zokaei and Husain 2019). These are areas of high unmet medical need. In particular, depletion of dopamine transmission (including D1 receptor signaling) in aging is known to contribute to age-related cognitive decline (Roth and Joseph 1994; Volkow et al. 1998; Karrer et al. 2017). Consequently, D1 receptor function has been the subject of extensive study at the behavioral, cellular, and molecular levels (Muly et al. 1998; Castner et al. 2000; Goldman-Rakic et al. 2000; Dunah and Standaert 2001; Castner and Williams 2007; Liu et al. 2017).

Development of D1 agonists to enhance cognition has presented a difficult challenge due to inverted U-shaped dose response (Williams and Goldman-Rakic 1995; Vijayraghavan et al. 2007). In addition, prolonged D1 stimulation by high affinity/long-acting agonists can lead to tolerance development (Asin and Wirtshafter 1993; Lewis et al. 1998; Gulwadi et al. 2001; Smith et al. 2002, Ryman-Rasmussen et al. 2007). Hence, clinical studies with full D1 agonists have had limited success (Blanchet et al. 1996; Giardina and Williams 2001; Zang et al. 2009). More recent approaches for D1 activation have been introduced including partial dopamine D1 agonists (Roberts et al. 2010; Balice-Gordon et al. 2020; Kozak et al. 2020; Riesenberg et al. 2020). An alternative approach would be to develop a positive allosteric modulator (PAM) for the D1 receptor, based on the hypothesis that the activity of a PAM would be dependent on endogenous dopaminergic tone, potentiating dopamine when and where it is released. This would offer a more physiological approach that could provide a better therapeutic margin, possibly avoiding the inverted U-shaped dose response and tolerance development seen with some D1 agonists. Recently, several selective D1PAMs from the tetrahydroisoquinoline structural class have been reported and characterized and pharmacological differentiation from D1 agonists was established in a series of in vitro and in vivo studies (Svensson et al. 2017; Bruns et al. 2018; Hao et al. 2019; Meltzer et al. 2019 and Svensson et al. 2019). Due to a species difference in D1 binding for the D1PAMs, these neurochemical and behavioral studies were performed in a transgenic mouse in which the murine D1 receptor was replaced with its human equivalent (hD1 mouse). Overall, the data supported a pro-cognitive potential for this approach (Bruns et al. 2018 and Meltzer et al. 2019). For the present study, we selected the D1PAM DPTQ (Hao et al. 2019), a close structural analog of the previously reported pharmacological tool DETQ and the clinical candidate LY3154207 (mevidalen; Hao et al. 2019; Wilbraham et al. 2021, Biglan et al. 2022 and McCarthy et al. 2022). DPTQ has an EC_50_ of 76 nM for potentiation of the dopamine-induced increase in cAMP in stably transfected human D1 cloned cells and possesses suitable physicochemical properties with acceptable brain penetration after systemic dosing (Hao et al. 2019).

Our main objectives with this study were to establish central pharmacodynamic activity and explore potential efficacy on cognition with a D1PAM in a higher species, the rhesus monkey. Members of the tetrahydroisoquinoline D1PAM series have been previously shown to have high affinity for this species, similar to humans (Svensson et al. 2017, Wang et al. 2018 and Hao et al. 2019). For initial pharmacodynamic testing, we selected the spontaneous eye blink rate model in the rhesus monkey to establish doses needed to achieve a central D1 response for DPTQ in a higher species. D1 agonists are known to increase eye blink rate in this model (Elsworth et al. 1991; Jutkiewicz and Bergman 2004). In addition, Parkinson’s disease patients have reduced spontaneous eye blink rate (Deuschl and Goddemeier 1998; Karson et al 1982). Although there are many interactive neurotransmitter systems involved in spontaneous eye blink rate, this model can be used as a marker for central dopamine D1 receptor activation (Jutkiewicz and Bergman 2004) and might also reflect effects on cognition (Taylor et al. 1999; Jongkees and Colzato 2016). A previous study with DETQ showed enhanced spontaneous eye blink rate, although with a smaller response than that which was observed with the D1 agonist SKF82958 (Bruns et al., 2018). For cognitive testing in the rhesus monkey, we selected the spatial delayed response task (Roberts et al., 2010), a highly established translational measure of spatial working memory that has been shown to engage the same neural circuitry in human and nonhuman primates (Jonides et al., 1993; Goldman-Rakic, 1996; McCarthy et al., 1996; Postle et al., 2000).

## Methods

### In vitro testing in the cAMP assay

The human, rhesus monkey, dog, or mouse D1 receptor was cloned into the Jump-In vector system (Life Technologies Corp, CA) and transiently transfected into HEK293 cells. Increases in cAMP by DPTQ (free base, Eli Lilly) were measured in the presence of an EC_20_ concentration of dopamine. The EC_50_ values for each species along with % max stimulation (where dopamine max stimulation equals 100%) are presented in Table 1. These in vitro studies were performed at Eli Lilly and Company, Indianapolis, in USA. For further details on the measurement of effects on cAMP accumulation in these transiently transfected cells, see Hao et al. (2019).

**Table 1:** D1 receptor potency of DPTQ in different species. Shown is the EC_50_ for potentiation by DPTQ of the increase in cAMP induced by an EC_20_ concentration of dopamine along with maximal stimulation of cAMP expressed as % of maximal full agonist (dopamine) response (*n* = 4)

Species	cAMP EC_50_ (nM)	SE	Max stimulation (% of max DA)	SE
Human	22.9	3.9	77	2.8
Dog	21.5	2.8	88	2.7
Rhesus	38	11	82	3
Mouse	534	156	41	1.7

### Spontaneous eye blink rate in the rhesus monkey

#### Animals

Eleven 5–6-year-old, male rhesus monkeys (Chengdu Ping, An Animal Breeding and Research Base, Sichuan Province, China) with an average weight of 4.83 kg were included in the study. The animals were randomly divided into three groups, DPTQ higher cumulative dose group (*n* = 4); DPTQ lower cumulative dose group (*n* = 3); and vehicle (20% Captisol (CyDex Pharmaceuticals, Inc., KS) in NaPO4 buffer) group (*n* = 4). The D1 agonist SKF82958 was included as a positive control; the results for this compound (not shown here) were recently published together with data for a different D1PAM (DETQ, see Bruns et al. 2018). The D1 selective antagonist SCH39166 hydrobromide (Cat No.: 2299, Batch No.: 3A/129719) was purchased from Tocris, MN, and was prepared in Millipore pure water with the injection volume 0.2 ml/kg. The D1 antagonist was dosed 15 min before vehicle or DPTQ (*n* = 4 animals per group).

The monkeys were housed individually in a climate-controlled (22–24℃) and humidity-controlled (40–70%) vivarium. A 12 h light/12 h dark cycle was in effect (light on from 7 am–7 pm). Monkeys had unlimited access to water and received a daily allotment of high-protein monkey chow, supplemented with fruit every day. Before compound administration and during test hour, monkeys were not fed with fruits or food.

The Spontaneous Eye Blink Rate study was performed at ChemPartner, Shanghai, China. The monkey housing in the Shanghai ChemPartner large animal facility is fully accredited by AAALAC. The animals were socially pair-housed in 2 interconnected cages, enabling visual and verbal communication with neighboring monkeys as well as self-viewing from a hanging mirror on each cage. The cage dimension for each monkey was 0.9 (*L*) × 0.9 (*W*) × 2 (*H*) m, with gourd, foraging ball, or ball chew provided inside. The enrichment program included television shows 2–3 times per week and background music for several hours every other day. Animal health status was checked periodically by the attending veterinarian and daily by the animal care staff for their (1) appearance, hair, and tail; (2) feces and urination; and (3) gross behavior, food consumption, and signs of illness. All training and testing protocols were approved by Institutional Animal Welfare IACUC guidelines and policy committee at ChemPartner (Shanghai, China).

#### Apparatus and experimental protocol

Spontaneous eye blinking rate was measured during observational experiments conducted in a specially constructed monkey chair (Mason et al. 2019). A U-shaped neck plate that helped to fixate the orientation of the head was used to facilitate observation of eye blink rate response. A compact video camera (Panasonic/wv-cp480/CH) on a tripod was positioned in front of the seated monkey. Two camera images were combined by video collector (Color QUAD system) and transferred to the EthoVision software (Noldus, Leesburg, VA) on the computer for on-line viewing and recording.

Two weeks before testing, monkeys were trained daily to sit in the monkey chair for two 2-h periods with food reward. During post-training test sessions conducted between 9:00 am and 4:30 pm every day, each monkey was studied for nine consecutive 15-min components. Each 15-min block consisted of a 10-min habituation period followed by a 5-min period during which eye blinking was counted. The video was displayed on a computer screen to allow continual observation of the subject. Observers blind to the treatment conditions later scored each 5-min videotaped session. An eye blink was defined as a visible, rapid opening and closing of the eyelid. Two observers independently confirmed the measurement with reliability > 90%.

#### Pharmacological testing

The effects of different D1 ligands on spontaneous eye blink rate were studied by a cumulative dosing procedure (Jutikiewicz and Bergman 2004). Active compound or vehicle (Captisol 20% w/v NaPO4 buffer) was administered after obtaining two baseline eye blink recordings. The ligand was given in random order to different monkeys of each group as described above. To study dose response and time course, incremental doses of DPTQ were administrated at the end of 15-min components of each test session.

#### Chemicals

DPTQ (free base, see Hao et al. 2019) was supplied by Eli Lilly Company. Captisol (Lot No.: NC-04A-05034) was prepared as follows: Captisol 20% w/v NaPO4 buffer 25 mM, pH 8. The injection volume for DPTQ was 0.5 ml/kg IM. DPTQ at 0.1 mg/kg, 0.5 mg/kg, and 1 mg/kg were dosed as clear solution, while the 5 mg/kg and 10 mg/kg doses were formulated as fine suspensions. The above vehicle solution was stored at 4℃ in a refrigerator and restored to room temperature before use. When preparing DPTQ fresh daily, a portion of the vehicle (20% Captisol) was added to the compound and stirred to wet, followed by addition of the remainder of the vehicle and mixing until the solution became homogeneous. The suspension was then sonicated on an ice bath for 40 min to reduce particle size. Animals were dosed while in the chair.

#### Data analysis for eye blink rate

Results for each monkey were expressed as the rate of eye blinking (blinks per minute) averaged over each 5-min testing epoch. The effects of vehicle and D1 PAM on eye blinking were averaged for each group of monkeys and expressed as mean ± SE. The effects of DPTQ and vehicle were analyzed with two-way ANOVA (D1 PAM and time) followed by Bonferroni post hoc test (GraphPad Prism, San Diego CA).

### Spatial working memory task

#### Animals

Sixteen rhesus and one stumptail macaque participated in the study (details provided below) that was performed at Yale University, New Haven CT, USA. Animals were used and cared for in full accordance with Yale University’s IACUC guidelines and polices in addition to all U.S. Federal policies and regulations. The monkeys were fed their normal diet each day, including appropriately nutritional biscuits and multiple fruits and vegetables for enrichment. Testing and any training occurred just before their main food course. This food consisted of biscuits, delivered each day, and all animals had full 24-h access to ad libitum water. Participation in cognitive testing was ensured by the enrichment of the environmental stimulation and the provision of the animals’ favorite food treats as rewards in the task, including yoghurt, raisins, almonds, grapes, fruit loops, and multiple other food items (Roberts et al 2010).

#### Cognitive testing

Spatial delayed response is an established translational measure of spatial working memory that has been shown to engage the same neural circuitry in human and non-human primates (McCarthy et al. 1996). The circuitry involved in this response is almost exclusively “dorsal stream” involving dorsolateral prefrontal cortex, particularly Area 46, and the lateral intraparietal sulcus (Goldman-Rakic 1996). For a full description of the task used here, see Roberts et al. (2010). Animals were tested in a sound-attenuated room incorporating a testing chamber. In this task one of several well locations is baited with food in view of the animal and the wells are then covered with identical plaques. An opaque shutter is then lowered for one of 5 variable delays and then raised to allow the animal to make a response to one of the well locations to retrieve the food. Each delay length was repeated 4 times in a semirandom distribution across the 20 trials in each testing session. Each animal was stabilized prior to commencing any administration by gradually incrementing the number of wells (starting at just 2) and the lengths of the delay. The 5 variable delay lengths were set at 0, 1, 2, 3, and 4 s multiplied by a factor “N.” Thus, N was incremented by 1 up to a maximum of 10 until the subject performed at 80% or more across 3 consecutive test sessions whereupon the number of wells was incremented by 1. This process was repeated for each individual until they reached stable performance (65–75% ±  ≤ 2.5% correct). This normalized level of performance allows for the sensitive detection of any impairment or improvement produced by experimental conditions. For baseline data for this study, we took 7 or more consecutive data points that had been collected in previous weeks which fell within the stable limits for the test. For the total group of 17 animals participating in this study, the median well number was 4 (range 2–7) and the median N value was 3 (range 1–7). Animals were typically tested 2 to 3 days a week.

#### Compound administration

DPTQ (free base, Eli Lilly & Co.) was prepared as described above for the spontaneous eye blink test. Animals (4 males and 6 females, mean age = 22.1 ± 1.7 years) were originally assigned to receive either vehicle or DPTQ 2.5 mg/kg IM in a semi-random design. One animal received vehicle but was not dosed with DPTQ. This same group of animals was later used in a repeated dosing study with DPTQ 2.5 mg/kg vs vehicle. After a protracted washout period of several months we tested the lower doses of 0.1 mg/kg and 1.0 mg/kg in ascending order. This group of 10 animals included 3 from the previous study (3 males, 7 females (including one aged stumptail macaque); average age = 22.6 ± 1.7 years). The youngest animal in this study was aged 17.3 years. None of the animals were naïve and had all been used in previous studies with other compounds. They were given an extensive washout of weeks or months prior to this study and were established to have normal baseline performance on the task. During the study, animals were washed out based on known PK properties (half-life) of this drug as well as the baseline performance of each individual animal. That is, performance was required to return to baseline prior to commencing any other condition and a washout period of at least two weeks was typically employed. Cognitive testing was conducted 1 h post injection to match the time of maximal effect observed in the original eye blink study.

#### Cognitive data analysis

The effect of administration of vehicle and each dose of DPTQ was analyzed using 1-way analysis of variance (ANOVA) followed by post hoc comparisons based on false discovery rate (FDR), using the two-stage step-up method of Benjamini et al. (2006; GraphPad Prism 9.1). This methodology was chosen considering the high number of groups (days) and the limited number of tests (animals). An *α* level of 0.05 was used to determine statistical significance. After initial testing to ensure no significant effects of vehicle (see “Results” below) comparisons were performed with baseline (averaged over several sessions) as the control.

### Plasma ligand exposure analyses

Plasma samples were collected in EDTA tubes at 0, 0.25, 0.50, 1, 2, 4, 8, 12, and 24 h after a bolus IM injection and stored at − 70C until further analysis by LC/MS (for further details see Hao et al. 2019). For the lower dose of 0.1 mg/kg, IM, plasma samples were collected from three of the animals participating in the spatial working memory task at Yale University, at approximately 1.5 h after dosing (~ 30 min after completion of behavioral test session). Animals were acclimated to sitting freely in a customized chair and having one leg held for hair trimming (if necessary), wiping with 70% alcohol, and saphenous venopuncture using a BD Vacutainer tube inside a safety holder. For the 5 mg/kg, IM dose, plasma samples were collected in a separate study in three male adult macaque monkeys at Eli Lilly and Company, Indianapolis, IN. The collection and storage of samples followed the procedure described above, and analysis of D1 ligand levels was carried out at Eli Lilly and Company, Indianapolis, IN, USA, using liquid chromatography and tandem mass spectrometry.

## Results

### Effects of DPTQ on cAMP accumulation in four species

DPTQ was tested in HEK293 cells transiently expressing human, dog, rhesus monkey, and mouse dopamine D1 receptors. Potentiator EC_50_ and Emax values for the dog and rhesus monkey D1 receptors were similar to those for the human D1 receptor (Table 1). However, the potentiator EC_50_ for the mouse D1 receptor was shifted rightward about 14- to 23-fold compared to the rhesus monkey and human respectively. In addition, the Emax for the mouse D1 receptor was only 41%, compared with 77–88% for the other three species.

### Spontaneous eye blink rate

**Fig. 1:**
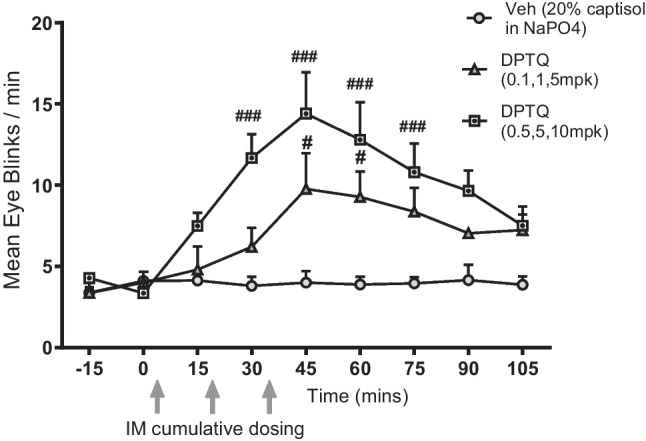
Time course of cumulative dose of DPTQ on mean eye blinks per minute in rhesus monkeys. Results are presented as the mean (for rates of eye blinking (DPTQ vs vehicle: #*p* < 0.05; ###*p* < 0.001; *N* = 3 DPTQ low dose, *N* = 4 monkeys all other dose groups)). Error bars represent standard error of the mean (SEM)

The mean baseline eye blink rates before dosing did not exceed 10 blinks/min, and the eye blink rates were relatively stable across test sessions within individual subjects after vehicle injection. DPTQ was tested in two cumulative dose regimens (Fig. 1). The higher cumulative dose of DPTQ (0.5 mg/kg, 5 mg/kg and 10 mg/kg, IM) resulted in the highest rates of eye blinking. The time course effect following these higher doses showed that significant increases of the post-DPTQ eye blink rate occurred at 30 min (*p* < 0.01), 45 min (*p* < 0.001), 60 min (*p* < 0.01), and 75 min (*p* < 0.05) (Fig. 1). The lower cumulative dose of DPTQ (0.1 mg/kg, 1 mg/kg and 5 mg/kg, resulted in small but significant (p < 0.05) increases in rates of eye blinking only at the 45-min and 60-min time points (Fig. 1). During recording and observation, some vehicle group monkeys fell asleep or became drowsy in the monkey chair as time went by. In contrast, all of the DPTQ-treated monkeys remained alert throughout the test period of close to 2.5 h.

Both high and lower cumulative doses of DPTQ resulted in some degree of oral movement such as tongue protrusion (data not shown). However, only the higher cumulative dose showed a clear effect at 30 min after DPTQ administration when compared to the vehicle group. This is consistent with an activation of tongue movement following D1 receptor stimulation reported in monkeys (Bédard and Boucher 1989).

**Fig. 2:**
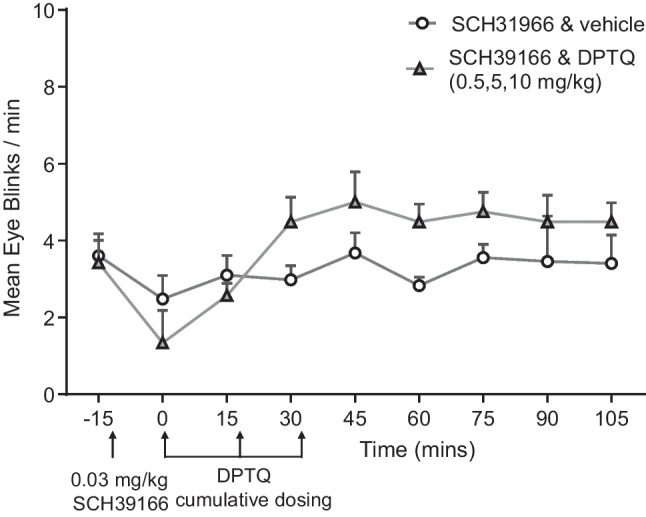
The D1 antagonist SCH39166 blocks the increase in spontaneous eye blink rate induced by high cumulative doses of DPTQ. Results are presented as the mean (± SEM) for rates of eye blinking (*n* = 4/group)

Administration of the D1 selective antagonist SCH39166 (0.03 mg/kg, IM) alone induced a brief reduction in eye blink rate, and visual observations indicated that the animals were slightly sedated. This effect lasted for about 15 min (Fig. 2). Animals pretreated with SCH39166 showed only a small, non-significant (*p* < 0.05) increase in eye blink rate induced by higher accumulated doses (0.5, 5, 10 mg/kg) of DPTQ (Fig. 2; compare to Fig. 1).

### Analyses of plasma levels of DPTQ in the monkey

**Fig. 3:**
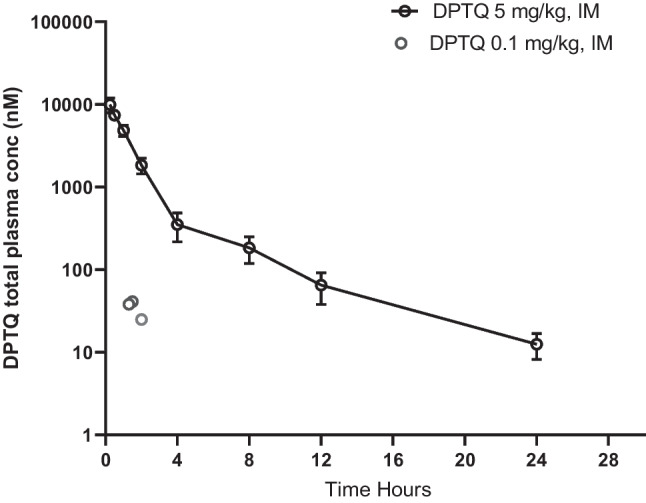
Total plasma levels (nM) of DPTQ after 5.0 or 0.1 mg/kg, IM. Plasma samples were collected for up to 24 h after 5 mg/kg, IM and at 1.5 h after dosing of 0.1 mg/kg, IM (*n* = 3 per time point in both studies). Error bars represent SEM

In the first study, plasma samples were collected at various time points from three animals for up to 24 h after a dose of 5 mg/kg IM (Fig. 7). High total plasma levels of DPTQ were measured for the initial 2 h ranging from about 9000 to 1800 nM with estimated unbound plasma concentrations of 674 to 125 nM. Based on data that DPTQ has an unbound brain vs unbound plasma concentration ratio of about 0.3 and unbound ligand fraction of 0.068 (J. Cramer, unpublished data), the projected unbound brain concentrations for DPTQ after 5 mg/kg injection would thus range from 202 down to 38 nM over the first 2 h post dosing. These concentrations are within the range of the hD1 EC_50_ value for DPTQ in the cAMP assay reported to be 76 nM (Hao et al. 2019) and at or above the monkey D1 EC_50_ of 38 nM reported here using transiently expressed cells (Table 1). In the second study, samples were collected after the low dose of 0.1 mg/kg, IM from three animals at ~ 1.5 h after dosing in the cognition study. As shown in Fig. 3, the low dose of 0.1 mg/kg IM resulted in about 50 × lower plasma levels of DPTQ. The total D1 PAM plasma concentrations ranged from 56 to 91 nM (Fig. 7). Using the same assumptions as above for unbound ligand fraction and unbound brain vs unbound plasma ratio, the estimated unbound ligand concentrations in the brain would be 1–2 nM at 1.5 h after the 0.1 mg/kg, IM dose. These levels are far below both the monkey and human EC_50_ values for potentiation of dopamine induced activation of cAMP in the vitro assay.

### Effects of DPTQ spatial working memory performance at an anticipated effective dose

**Fig. 4:**
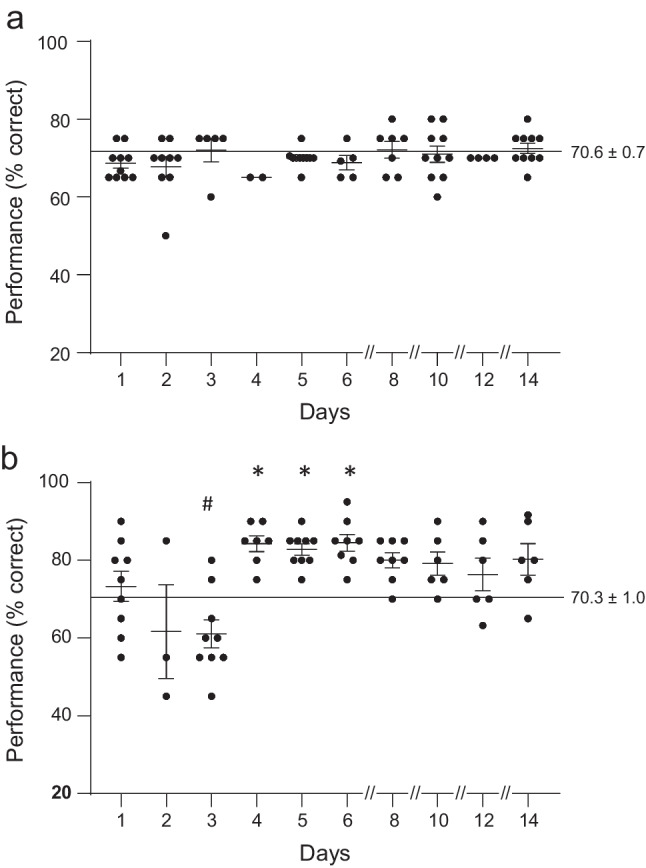
Acute and delayed effects of DPTQ administration at 2.5 mg/kg IM in comparison to vehicle. **a** Vehicle given on day 1 produced no significant changes in cognitive performance over 14 days, while **b** a dose of 2.5 mg/kg IM produced a complex time-dependent effect involving a delayed decrease followed by an increase in scores (*p < 0.01, #*p* < 0.05 in comparison to baseline). Individual scores are shown together with corresponding mean and standard error bars for the group in these performance figures. Horizontal cursors indicate baseline performance measured over several test sessions prior to DPTQ administration

A single dose of DPTQ was originally tested at 2.5 mg/kg in comparison to vehicle. Separate but overlapping baselines were used for the two conditions. Vehicle data are shown in Fig. 4a for a group of 10 animals. Group changes in performance varied little from baseline over the next 2 weeks with the largest deviation for the group average reaching only 5% (*p* = 0.51, paired t-test within subject in comparison to baseline). Of note, no impact was seen on performance acutely on day 1. DPTQ (2.5 mg/kg, IM) data are shown in Fig. 4b for a group of 9 animals. 1-way ANOVA revealed that the total dataset for each animal for each day varied significantly from the baseline data for each animal (*F*[10,69] = 6.48, *p* < 0.0001). No immediate acute effect was seen on day 1 but, a slight (10%) diminution of performance was evident by day 2 which became more robust by day 3, more than 48 h post injection (mean = 61.1; post hoc comparison with baseline, *p* = 0.025). Note that in this first data set those tests on the days following day 1 were originally purely exploratory and therefore have low N values in some cases.

Overt behavioral observations suggest that the animals initially on days 2 and 3 were slightly activated by this dose including hyper-vigilance/attention, but not motoric activation, that could affect their response. This apparent impairment in cognition was immediately followed by a substantial improvement observed on the next day (mean = 84.3*)* that persisted for several days (days 4, 5, and 6, *p* < 0.01).

### Effects of repeated dosing on spatial working memory

**Fig. 5:**
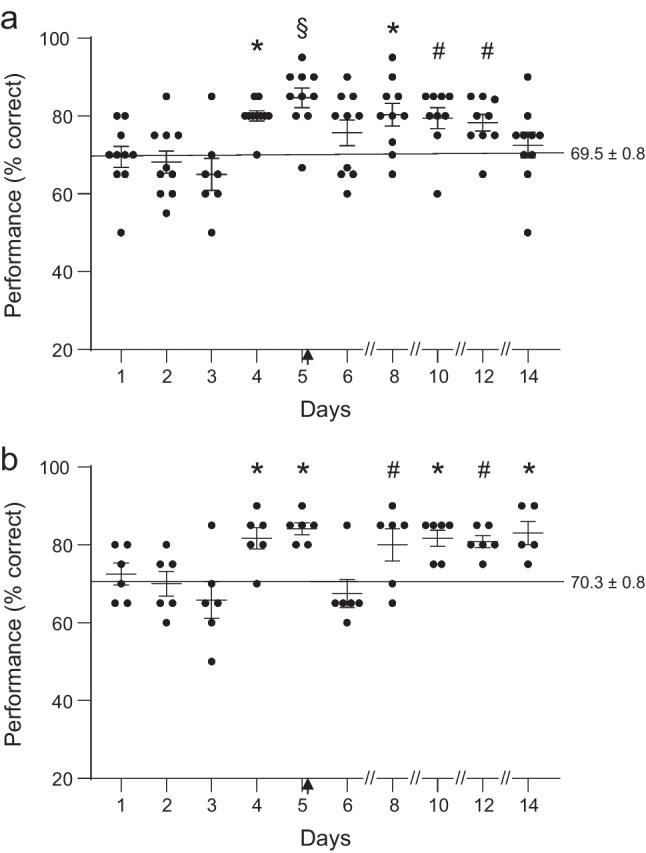
Comparison of the effects of administration of vehicle and a dose of 2.5 mg/kg on day 5 after prior dosing on day 1 with 2.5 mg/kg, DPTQ, IM. **a** Robust enhancement was again seen on days 4 and 5 which showed some persistence in the following week after vehicle was administered on day 5. **b** The same robust enhancement was seen after this acute dose but a decrease in performance to baseline levels was seen the day after the second DPTQ dose. Despite this decrease, performance returned to an elevated level over the next week (#*p* < 0.05, *p < 0.01, §*p* < 0.0001; vertical arrows indicate time of second injection)

In order to examine whether a repeated dose would result in tolerance, summation, or sensitization of these effects, a dose of 2.5 mg/kg was administered acutely on day 1 (1 h prior to testing and then either vehicle (Fig. 5a) or an additional dose of 2.5 mg/kg (Fig. 5b) was administered on day 5 (immediately post testing). Animals were randomly assigned to either DPTQ on day 1 and vehicle on day 5 (vehicle group) or DPTQ on both days (DPTQ group), then crossed over to the other treatment group after a suitable washout period The effects of administering vehicle as the second dose can be seen in Fig. 5a (*n* = 10). Except for vehicle administration on day 5, this is an identical experiment to the single dose study for DPTQ described above, as shown in Fig. 4. Results appear similar to those seen for the previous single dose study (*F*[10, 94] = 5.23, *p* < 0.0001), except that the deficit on day 3 was small and did not achieve significance (*p* = 0.278 vs baseline) after which an enhancement appeared over the next few days (days 4 and 8, *p* < 0.001; day 5, *p* < 0.0001; days 10 and 12, *p* < 0.05 vs baseline). A subgroup of the same animals (*n* = 6) were later assigned to the DPTQ group, receiving a repeated dose of 2.5 mg/kg DPTQ on day 5 (overall statistical significance *F*[10, 54] = 5.41, *p* < 0.0001). Following the acute dose of 2.5 mg/kg IM on day 1, a non-significant dip in performance was seen on day 3 which was followed a rebound enhancement on days 4 and 5 (*p* < 0.01). However, following the repeat of the dose on day 5, there is a reduction in performance to baseline on day 6, followed by enhancement for the subsequent week (days 8 and 12, *p* < 0.05; days 10 and 14, *p* < 0.01). Notably, performance was now significantly improved on the last day.

### Effects of low doses DPTQ at 0.1 and 1.0 mg/kg

**Fig. 6:**
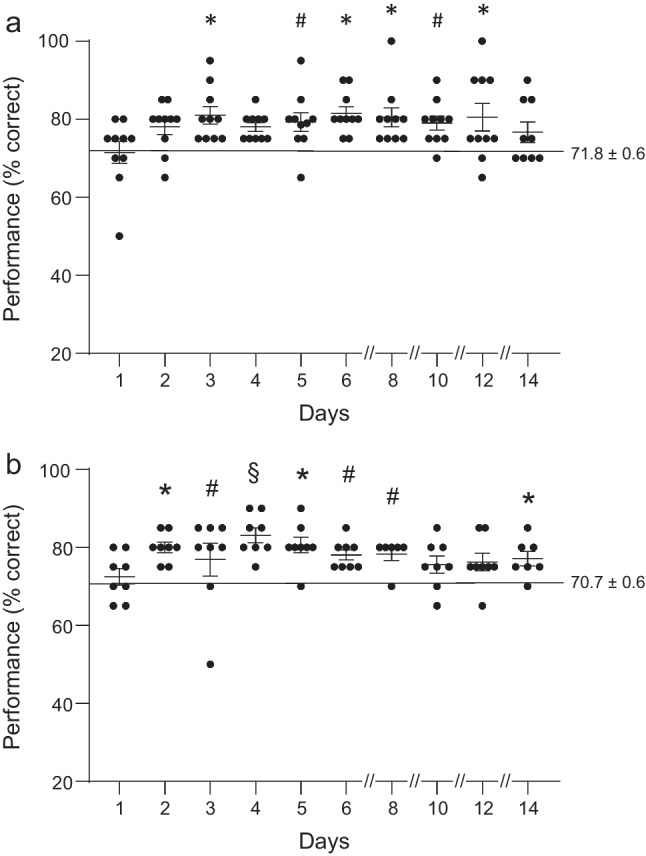
Effects of DPTQ on spatial working memory at 0.1 mg/kg and 1.0 mg/kg. **a** The low dose (0.1 mg/kg) produced a modest enhancement in performance over several days following the day of injection. **b** The intermediate dose of 1.0 mg/kg produced significant increases in performance which was particularly evident on day 4 (#*p* < 0.05, *p < 0.01, §*p* < 0.0001)

Based on the above data we tested the hypothesis that the dose of 2.5 mg/kg might be supra-optimal for beneficial effects on cognition, and that a lower dose might achieve a significant, enduring enhancement in working memory while still avoiding the induction of an early temporary deficit. As hypothesized, lower doses (1.0 and 0.1 mg/kg, IM) of DPTQ appeared to show enhancement without an early impairment of spatial memory (Fig. 6).

At the dose of 0.1 mg/kg (Fig. 6a), DPTQ appeared to produce no diminution of performance on day 3 but still a modest enhancement of working memory that lasted nearly 2 weeks in this group of 10 animals (*F*[10, 98] = 2.39; *p* = 0.014). In fact, a significant enhancement in cognition was observed on day 3 (*p* < 0.01) following a close to significant performance increase on day 2 (*p* = 0.053). While performance on day 4 again approached significance (*p* = 0.053), the following days showed clear enhancement (days 5 and 10, *p* < 0.05; days 6, 8 and 12, *p* < 0.01) up until day 14. For 8 of these animals then tested at the dose of 1.0 mg/kg, we again observed a significant improvement in working memory over the two week period (Fig. 6b; *F*[10, 74] = 2.75, *p* = 0.006). Interestingly a clear enhancement could be seen from day 2 (*p* < 0.01) and although performance on day 3 was still elevated (*p* < 0.05) there was a noticeable reduction is score that day before the strong improvement seen on day 4 (*p* < 0.0001). This enhancement continued for the next 3 days (day 5, *p* < 0.05; days 6 and 8, *p* < 0.05) and then faded until day 14 (*p* < 0.05). In order to make a direct comparison between doses we performed an ANOVA across the 3 doses for days 3, 4, and 5. When we compared the effects of each on day 3, where we saw the initial reduction in performance at the high dose, we found a critical effect of dose (*F*[2,23] = 10.18, *p* < 0.001). Both doses of 0.1 and 1.0 mg/kg yielded a significantly higher score than that at 2.5 mg/kg (*p* < 0.01, *p* < 0.05, respectively). Conversely, by day 4, a significant proportion of the variance in the data was also attributable to dose (*F*[2,22] = 4.547, *p* < 0.05) but this effect was inverted with the score at 2.5 mg/kg now becoming significantly greater than that at 0.1 mg/kg. No difference in scores could be attributed to the difference in dose on day 5.

### Dose-dependent effects in relation to delay duration

**Fig. 7:**
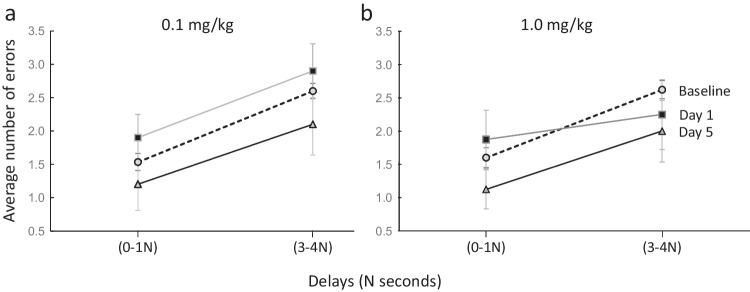
Delay-dependent effects of DPTQ at 0.1 and 1.0 mg/kg on days 1 and 5 in comparison to baseline. **a** The mean number of errors showed an increase in absolute values at both short and long delays after acute injection but this converted into modest improvements evident on day 5 (*n* = 10). **b** The intermediate dose of 1.0 mg/kg also showed no improvement acutely but induced an apparent decrease in errors at both the short and long delays observed on day 5 (*n* = 8). Error bars represent SEM

The same data was analyzed to examine the dose-dependent impact of the D1PAM treatment on delay dependent errors in the task and how this varied by time relative to administration of each dose of DPTQ. For this purpose, we chose to compare the time point of day 5 to compare with those errors seen after acute injection and baseline as the effects DPTQ appeared to be the most consistent across all doses on that day. No significant effects of any condition on day 1 were evident using 2-way ANOVA. As shown in Fig. 7, DPTQ at the low dose of 0.1 mg/kg produced a slight increase in errors at both short (0, 1) N and long (3, 4) N delays in the task when tested for its acute effects on day 1. However, 96 h later on day 5, this dose of the D1 PAM led to a small but non-significant decrease in errors, particularly at the long delays in the task. Two-way RMANOVA found no significant effect of dose and no interaction with delay on day 5. At the dose of 1.0 mg/kg, DPTQ produced no reduction in errors at short delays on day 1 but did produce a reduction on day 5. Although there was some evidence of a decrease in errors at the long delays on day 5, this was found to be non-significant probably due to the large variance in errors by delay in the task.

## Discussion

Positive allosteric modulators of the dopamine D1 receptor may allow for pharmacological support of D1 signaling potentially without the disadvantages such as an inverted-U response, tolerance, and tachyphylaxis associated with some D1 agonist treatments. In transgenic hD1 mice, newly disclosed D1PAMs such as DETQ and mevidalen have already been shown to live up to this promise in models such as locomotor activity and novel object recognition (Svensson et al. 2017; Bruns et al. 2018; Hao et al 2019, and Meltzer et al. 2019). However, the potential cognitive value of D1PAMs goes beyond the freedom from these limitations. Unlike full and partial agonists, D1PAMs cannot operate independently of dopamine transmission. For their action at the D1R, dopamine must be released at those specific sites and the degree of positive modulation should be dependent on the degree of dopaminergic stimulation at those sites, such as on the spines of pyramidal neurons (Smiley et al. 1994) or the dendrites of inhibitory interneurons (Muly et al. 1998) in regions such as PFC. This implies that D1PAMs can provide functional support of ongoing D1 signaling without contravening spatiotemporal barriers in the distribution of that signaling, acting in tandem with real-time dopaminergic stimulation of the orthosteric site. Such a biologically appropriate action may provide benefits for the treatment of disorders with insufficiency in D1signaling, such as age-related cognitive decline. Moreover, the availability of D1PAMs such as DETQ, DPTQ, and mevidalen may provide tools to unravel the multiplicity of mechanisms involved in the transition between deficient, optimal, and excessive D1R stimulation and its impact on neuroplasticity and cognition (Williams and Castner 2006). The findings of the present study demonstrate the significance of D1PAMs, not only for the treatment of neuropsychiatric disorders but also as pharmacologic tools for the dissection of the neuronal mechanisms underlying both the potential benefits and detriments to optimal cognitive function (Goldman-Rakic et al. 2004; Roberts et al., 2010; Svensson et al., 2019).

DPTQ together with the closely related structural analogs DETQ and mevidalen belong to the tetrahydroisoquinoline series of D1PAMs (Svensson et al., 2017 and Hao et al., 2019). These molecules show high potency and specificity for the human D1 receptor vs other targets including the dopamine D2 receptor. The lack of affinity of DPTQ for the D2 receptor was confirmed in a broad screening study using ^3^[H]-raclopride as ligand where the D2 was found to be Ki > 5.6 µM (unpublished findings). This is in line with previously published data for mevidalen and DETQ that showed no activity at 10 µM in functional and binding studies for the D2 receptor. DPTQ has significantly lower potency and efficacy at the native rodent D1 receptor. This is related to a rodent-specific mutation in the second intracellular loop where these molecules bind (Wang et al., 2018). As shown previously, these D1PAMs have similar potency and efficacy at non-rodent species including human, dog and rhesus monkey (Svensson et al., 2017, Wang et al., 2018, and Hao et al. 2019). For DPTQ, there is approximately a 40-fold higher potency for the human and monkey vs the mouse D1 receptor (see Table 1). As all the initial in vivo characterization of the D1PAMs was done in the humanized D1 knock-in mouse, we considered the monkey as an appropriate higher species for further pharmacological evaluation of this new mechanism.

### DPTQ acts dose-dependently on native primate D1R to alter eye blink rate

The initial experiments ascertained that DPTQ could affect the brain function in the nonhuman primate by a direct action at native D1R by observing its effects on eye blink rate and determined the relationship between this pharmacodynamic response and the pharmacokinetics of plasma exposure after IM administration. Since this the first D1 PAM study in NHP, we used a pharmacokinetic (PK) study and in vitro projections of unbound brain concentrations for the dose selection (5 mg/kg, IM). The projected unbound brain concentrations of DPTQ after 5 mg/kg, IM DPTQ ranged from 202 nM down to 38 nM over the 2 h post dosing. This correlated with eightfold down to onefold concentration of the DPTQ EC_50_ in hD1 and monkey D1 cAMP assay. Based on this, the 5 and 10 mg/kg doses should be enough for the in vivo study. This was also confirmed in the eye blink rate study. Furthermore, these data correlate from findings in both the humanized D1 mouse (Svensson et al. 2017; Hao et al. 2019) and healthy volunteers (Wilbraham et al. 2021) where we have seen that doses of D1PAM that generate unbound brain/CSF concentrations at, or greater than, the EC_50_ in the human D1cAMP assay also results in significant behavioral activating effects such as locomotor stimulation and wake-promotion.

In primates, including human, eye blink rate has been shown to be associated with attention, cognitive engagement and performance of complex behaviors (Jongkees and Colzato 2016; Ranti et al. 2020; Andreu-Sánchez et al. 2021; Dave et al. 2021). Central dopaminergic inputs to the frontal cortex and basal ganglia modulate voluntary and spontaneous eye blink mechanisms during cognitive processes (Maffei and Angrilli 2018; Pajkossy et al. 2018; Imburgio et al. 2021). Multiple studies have shown that human and monkey eye blink rate is increased by selective direct D1 receptor stimulation, while the role of a D2 receptor mechanism in monkey eye blink is less clear (Kleven and Koek 1996; Czoty et al. 2004; Jutkiewicz and Bergman 2004; Desai et al. 2007; Dang et al. 2017). In fact, Kotani et al. (2016) demonstrated that dopamine acts specifically through the D1 receptor to influence eye blink rate, not the D2 receptor family. In the present study, nonhuman primates showed a clear increase in spontaneous eye blink rate following cumulative dosing of DPTQ, consistent with a similar effect reported earlier with a D1 agonist SKF82958 and the D1PAM DETQ (Bruns et al 2018). The increase in eye blink rate by D1 PAMs monkeys therefore suggests a potential D1 mechanism in enhancing cognitive process (see “Spatial working memory” section next).

We observed that vehicle control monkeys became sleepy on the monkey chair during the course of the eye blink study, while monkeys receiving DPTQ were all highly alert and vigilant, especially in response to the higher cumulative dose. EEG measurements in transgenic hD1 mice have previously shown that central dopamine D1 receptors have an important role in behavioral wakefulness and vigilance (Qu et al. 2008; Herrera-Solis et al. 2017; Bruns et al. 2018). This D1-dependent vigilant state is presumably a pre-requisite to effective cognitive processes engaged in learning and working memory (Dai et al. 2020; Kozak et al. 2020; Zhang et al. 2019). This is consistent with the notion that cognitive enhancement, particularly that involving dopamine and D1 receptors, may extend to cognitive motivation, incentive intent (Carli et al. 1989) and behavioral effort. A clinically relevant example of this translation from cognition to action has been demonstrated in patients with Parkinson’s disease who show a significantly higher level of cognitive motivation, resulting in increased behavioral effort in “ON” as opposed to “OFF” states (McGuigan et al. 2019).

High doses of selective D1 receptor stimulation have been reported to cause spontaneous tongue protrusion in monkeys (Bédard and Boucher 1989; Koshikawa et al. 1991; Tomiyama et al. 2012). At the highest cumulative IM doses of DPTQ, oral movement with spontaneous tongue protrusion was observed in some monkeys in the present study. However, such oral movement with tongue protrusion was not reported with the closely related D1PAM mevidalen in recent clinical studies of adult human volunteers (Wilbraham et al. 2021) suggesting that such potential effects may not be relevant to humans.

The high plasma exposures for DPTQ after the 5 mg/kg, IM dose suggests potential brain levels that meet or exceed the EC_50_ value for the human or monkey D1 receptor in the cAMP assay for potentiation of dopamine. The increase in eye blink response at this dose is clear evidence of central D1 receptor activation which was also supported by overt observations of increased alertness compared to vehicle treated animals. Overall, these findings are in line with results from the hD1 mouse, in which D1PAMs have consistently shown behavioral activation with locomotor stimulation at doses that produce unbound brain concentrations at or above the in vitro hD1 EC_50_ level in the cAMP assay (Svensson et al. 2017 and Hao et al. 2019). As shown here projected unbound brain concentrations much greater than the monkey EC_50_ for cAMP are achieved after the dose of 5 mg/kg, IM but not the lowest dose of 0.1 mg/kg, IM. As discussed below, this suggests that cognitive enhancement occurs at doses (brain concentrations) that are significantly lower than what is required for motor stimulation and, indeed for stimulation of cAMP production. There are multiple examples of such ultra-low dose effects of D1 agonists in the literature (Cai and Arnsten 1994; Castner et al. 2000; Roberts et al. 2010) and nicotinic Alpha7 agonists (Castner et al. 2011). These effects are suggestive of a highly sensitive mechanism acting directly at certain synaptic sites and which then go on to influence synaptic plasticity which may involve immediate early gene expression.

### Dose-dependent effects of DPTQ on working memory in the primate

The results show that there was an entirely null acute effect of DTPQ on spatial working memory 1 h after injection, regardless of the dose administered. This was despite the fact that we can infer that some neuronal influence was occurring at the 1 h mark post administration from the observed effects on eye blink rate. The pharmacokinetics clearly demonstrates that was virtually no remaining plasma exposure beyond the first day. However, many neuro-active agents have effects that last days or weeks after exposure (for example, the antidepressant activity of ketamine). The present study found that DPTQ did indeed have a strong significant action on working memory — a substantial working memory impairment observed 49 h after IM administration at a dose of just 2.5 mg/kg. This dose would have been definitively expected to induce an acute elevation of eye blink rate but did not induce the cognitive deficit for another 48 h. Indeed, there is one study indicating that eye blinks may have a deleterious effect on working memory (Irwin 2014), although the doses used here would not be expected to have a dramatic effect on eye blink rate, certainly not 49 h later. Moreover, a recent study has shown that human subjects’ spontaneous eyeblinks during delay periods of working memory was strongly correlated with their spatial working memory performance (Ortega et al. 2022). In addition they found an association of increased eyeblinks during both stimulus encoding and retrieval during the task, potentially reflecting elevated visual attention. As such, it can reasonably be assumed that some long-term mechanism of neuroplasticity must have been involved in the generation of cognitive impairment. The implication of neuroplasticity in the effects of DPTQ at this dose was then made all the more certain by the fact that just one day later performance on the task became substantially improved and this cognitive enhancement appeared to persist for the following week. We further examined these neuroplastic effects DPTQ on cognitive function by administration of repeated doses on day 1 and day 5. On examination of the effect of repeated dosing, it was evident that the reduction seen in performance on day 3 after the initial dose in the prior experiment was no longer significant which might suggest some sustained effects in the mechanism of action involved. Despite this result, it was observed that a second, repeated dose of DPTQ at 2.5 mg/kg on day 5 did produce a stark impairment in performance the very next day which resolved within the next two days to yield a significant improvement in performance of the entire course of the following week. Thus, despite remaining effects on day 3, repetition of this dose on day 5 may actually have led to some summation of its beneficial actions.

We then attempted to repeat the cognitive enhancement without inducing a prior cognitive deficit using even lower doses of 0.1 and 1.0 mg/kg. What we observed is that we not only achieved a significant level of cognitive enhancement but this now occurred just 1 or 2 days after DPTQ administration without any intermittent impairments on performance in the task. Although this enhancement was not as dramatic as that shown for the dose of 2.5 mg/kg, it still appeared to persist over the next several days. Notably, we again saw signs of a decrease in performance on day 3 at the intermediate dose of 1.0 mg/kg.

These findings across all dosing regimens clearly implied that a mechanism of long-term neuroplasticity was involved and although this may have been amplified by a process that induced a temporary deficit, it was not entirely dependent upon it.

There are a number of neuronal mechanisms and signal transduction pathways that may have been involved in these effects. Although plasma concentrations of DPTQ are negligible after 12 or 24 h, it is possible that some binding to the intracellular allosteric site may have persisted for considerably longer and this may have eventually led to desensitization and/or internalization of D1 receptors and a consequent period of deficient D1 signaling. However, there is no evidence of this and it is unlikely, given the moderate potency of DPTQ for the allosteric binding site (Hao et al. 2019). While there is some evidence that certain D1 agonists my affect PKC signaling at higher doses (Lee et al. 2014; Glovaci and Chapman 2015), a more likely signaling route seen at the low doses tested here might be through ERK1 signaling arising from D1 facilitated recruitment of beta-arrestin (see Yang 2021 for review). It has been shown that, under conditions of dopamine depletion, there is an important shift in D1R signaling to a route that occurs through extracellular signal-regulated kinase/mitogen-activated protein kinase (ERK1/2/MAP kinase; Gerfen et al. 2002; Haberny et al. 2004; Papadeas et al. 2004). In a study of rodent PFC, exposure to novel objects produced an immediate increase of phosphorylated ERK1/2. Inhibition of ERK kinase impaired long-term memory for the object 24 h later but not the “shorter” term memory 1 h after initial exposure (Nagai et al. 2007). ERK1/2 phosphorylation in vivo and in vitro was increased by administration of SKF38393 and long term memory was impaired by direct injection of SCH23390 but not raclopride directly into PFC. In unilateral 6-OHDA lesioned rat slices, antagonists of PLC, PKC, and IP3 receptors dramatically reduce the ability of SKF38393 to induce the activation of ERK1/2 (Fieblinger et al. 2014). These findings provide the strong suggestion that, under conditions of dopamine deficiency, D1R stimulation may be more capable of signaling through the ERK1/2 pathway and having a more powerful influence on protein synthesis, synaptic plasticity, and long-term memory, of the kind that might explain the delayed effects of DPTQ on working memory elucidated in this study arising from local translation in dendrites of prefrontal neurons (Sutton and Schuman 2005).

It is possible that there are multiple mechanisms involved in the effects of DPTQ at the dose of 2.5 mg/kg. One that is responsible for the early decrement in working memory performance and another that accounts for the following enhancement. Thus, it is conceivable that the strength of this beneficial effect on neuronal plasticity may have been dependent on the strength of the original potentiation of D1 signaling but could still occur even if that potentiation was not so strong as to result in an impairment of cognition. Therefore, particularly in relation to the initial results with this dose, the possibility exists that the inverted-U hypothesis of D1R dependent dopamine signaling may not just be a static phenomenon but may also have important temporal components. Indeed, these temporal components may be highly significant for the treatment of motor vs. cognitive disorders. In addition, what our data suggests is that the inverted U-shaped like phenomenon at high doses may not be a significant disadvantage with this mechanism as precognitive effects were observed over a wide dose-range (at least 25-fold). This could also apply to D1 agonists as discussed below.

A significant impact of the facilitation of D1R signaling on mechanisms of plasticity involved in cognition is consistent with our previous studies in the nonhuman primate. Thus, we have shown that repeated bouts of an ultra-low dose of the full D1 agonist ABT-431 could result in a long-term reinstatement of normal working memory performance in animals being treated with chronic haloperidol to produce a down-regulation of D1R (Castner et al. 2000). The same regimen produced an improvement in working memory in aged rhesus monkeys that lasted for up to a year and could be reinstated later by a repetition on the same regimen (Castner and Goldman-Rakic 2004). The mechanisms of neuroplasticity involved must be functionally specific, potentially encompassing the development of dendritic spines and the recruitment of AMPA and NMDA receptors to the postsynaptic membrane on those spines (Buonarati et al. 2019; Delint-Ramírez et al. 2008; Mahan et al. 1990; Neve et al. 2004). We have recently shown that the D1PAM DETQ enhances brain levels of both pCREB and the AMPA receptor pGluR1 in the hD1 mouse, similar to what was observed for the D1 agonist SKF82958 (Bruns et al. 2018), providing further neurochemical evidence for enhanced synaptic plasticity. Further research is required to investigate the role of this mechanism in the long term effects on cognitive enhancement induced by D1PAMs such as DPTQ.

When we examined the delay-dependent effects of low and intermediate doses of DPTQ we found that the cognitive enhancement applied to both the short and long delays that individual animals experienced in the task by day 4. This finding is consistent with the evidence that D1 signaling is involved in both the induction of persistent activity in prefrontal pyramidal cells that may represent the working memory buffers involved (Durstewitz et al. 2000). However, this only reached significance for the effect of the 1.0 mg/kg dose at short delays which raises the question of how far any of the hypervigilance and increased attention was more observable at the higher dose may have contributed to the enhanced cognition. Regardless of the actual mechanisms involved it would appear that the combined reduction in the number of errors at both short and long delays contributed to the improvements in working memory.

The present study establishes that the D1PAM DPTQ has prominent biological actions resulting from facilitation of native D1 receptors in the nonhuman primate. These findings are a major step forward to further understand the D1PAM mechanism and potential as they provide the basis for translational research not previously possible, beyond the employment of humanized D1 knock-in mice. While clearly observable motor effects occurred at doses of 5–10 mg/kg IM, in the eye blink test, both long-term beneficial and very short-term deleterious effects of DPTQ on the spatial delayed response task were seen in a lower dose range, extending down to just 0.1 mg/kg. The low plasma levels of DPTQ at the 0.1 mg/kg dose with projected low unbound brain exposures support the lack of visible motor effects at this dose that could be deleterious on cognition. Given the fact that exposure at this dose would fall well below the EC_50_ for the accumulation of cAMP, it appears that alternative signal transduction pathways are likely to be involved in its effects on cognition at this dose. This finding is consistent with the literature suggesting that ultra-low doses of D1 agonists may be optimal for improving working memory and points to the potential therapeutic benefit of a D1PAM over full and partial D1 agonists (Cai and Arnsten 1997; Castner et al. 2000; Castner and Goldman-Rakic 2004; Kozak et al. 2020). These results demonstrate that cognitive enhancement with this D1PAM can be achieved without the induction of any incumbent deficit when low doses of DPTQ are used. It remains to be seen whether repeated dosing of the D1 PAM at 0.1 or 1.0 mg/kg can induce a sustained enhancement of cognition that potentially can be of therapeutic benefit for this mechanism. Taken together, the behavioral endpoints show several potential advantages of the D1PAM (DETQ), including lack of inverted U-shaped dose–response and rapid tolerance development. These findings were also confirmed with the close structural analogs mevidalen/LY3154207 (Hao et al. 2019) and LY314885 (Hao et al. 2022). We conclude that DPTQ and similar structural analogs may have profound benefits for the treatment of cognitive deficits in several neuropsychiatric disorders including cognitive decline due to aging or neurodegeneration.
